# Cement augmentation of an angular stable plate osteosynthesis for supracondylar femoral fractures - biomechanical investigation of a new fixation device

**DOI:** 10.1186/s12891-020-03215-3

**Published:** 2020-04-11

**Authors:** Martin Bäumlein, Antonio Klasan, Christine Klötzer, Benjamin Bockmann, Daphne Eschbach, Matthias Knobe, Benjamin Bücking, Steffen Ruchholtz, Christopher Bliemel

**Affiliations:** 1 Center for Orthopaedics and Trauma Surgery, University Hospital Giessen and Marburg, Location Marburg, Marburg, Baldingerstrasse, 35043 Marburg, Germany; 2 Department of Orthopaedics and Trauma Surgery, St. Josef Hospital, Ruhr-University Bochum, Bochum, Germany; 3 Department of Orthopedics and Trauma, Lucerne Cantonal Hospital, Luzern, Switzerland

**Keywords:** Distal femoral fracture, Polyaxial angular stable plate osteosynthesis, Cement augmentation, Osteoporosis, Biomechanical analysis, Fenestrated screws

## Abstract

**Background:**

Implant anchorage in highly osteoporotic bone is challenging, since it often leads to osteosynthesis failure in geriatric patients with supracondylar femoral fractures. Cementation of screws is presumed to prevent such osteosynthesis failure. This study aimed to investigate the effect of a newly designed, cementable fenestrated condylar screw for plate fixation in a biomechanical setting.

**Methods:**

Eight pairs of osteoporotic cadaver femora with an average age of 77 years, ranging between 62 and 88 years, were randomly assigned to either an augmented or a non-augmented group. In both groups an instable 33-A3 fracture according to the AO / OTA classification was fixed with an angular stable locking plate. All right samples received a cement augmentation of their fenestrated condylar screws with calcium phosphate bone cement (CPC). Mechanical testing was performed at a load to failure mode by cyclic axial loading, using a servohydraulic testing machine.

**Results:**

With a mean of 2475 N (95% CI: 1727–3223 N), the pressure forces resulting in osteosynthesis failure were significantly higher in specimen with cemented condylar screws as compared to non-cemented samples (1875 N (95% CI: 1320–2430 N)) (*p* = 0.024). In both groups the deformation of the constructs, with the distal screws cutting through the condylar bone, were the most frequent cause for failure. Analysis of axial stiffness (*p* = 0.889) and irreversible deformity of the specimens revealed no differences between the both groups (*p* = 0.161). No cement leakage through the joint line or the medial cortex was observed.

**Conclusion:**

Based on the present study results, the newly introduced, cementable condylar screw could be an encouraging feature for the fixation of supracondylar femoral fractures in patients with reduced bone quality in terms of load to failure accuracy of the cement application.

## Background

Osteoporotic fractures remain an unsolved problem in trauma and orthopaedic surgery, representing an immense burden for western industrial health care systems. Because of an ongoing demographic change with more and more active, old aged people, an increasing amount of distal femoral fractures will occur [1]. An osteoporotic bone structure often contributes to the occurrence of such distal femoral fractures in these patients [2, 3]. To improve fracture fixation in the old aged, polyaxial, angular stable implants as well as new, minimally invasive operation techniques have been developed.

Notwithstanding such innovations in implant technology and soft tissue management, the fixation of far distally located femoral factures still remains problematic, especially in severely osteoporotic bone [4]. Therefore, in the last few years, cement augmentation of implants gained more and more popularity to improve implant anchorage.

**Fig. 1:**
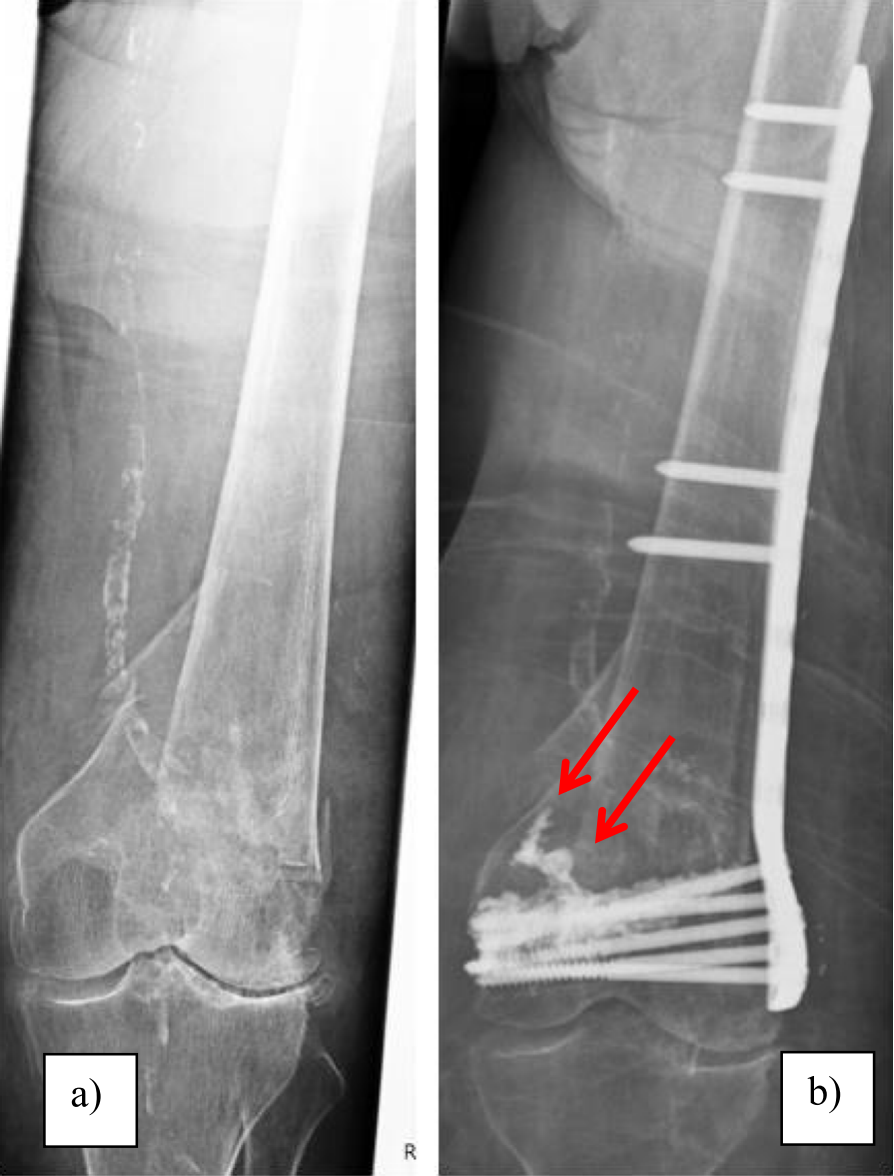
Clinical case of a 93-year-old woman with highly osteoporotic bone structure and the presence of a supracondylar distal femoral fracture (**a**). Fracture fixation was conducted with a plate osteosynthesis and cement augmentation of the condylar screws. As shown (red arrows), an uncontrolled cement outflow towards proximal occurred (**b**)

The expected, positive effects of cement augmentation on implant anchorage in osteoporotic distal femoral fractures have previously been investigated [5, 6]. Nevertheless, the uncontrolled extrusion of bone cement into the surrounding soft tissue or into the joint line still poses a significant risk of this surgical technique. In this context, Fig. 1a presents the clinical case of a 93-year-old woman with a supracondylar distal femoral fracture and an associated highly osteoporotic bone structure. Fig. 1b shows the fracture fixation via minimally invasive inserted plate osteosynthesis and cement-augmented condylar screws. While proper fracture fixation was achieved nevertheless an uncontrolled cement outflow towards proximal (red arrows) occurred (Fig. 1b).

As a further step to previous research [5, 6], the present study focused on a safer and more suitable cement augmentation technique, resulting in the development of a fenestrated condylar screw, suitable for the cement augmentation of angular stable locking plates.

To analyse the value of this new fixation device, the aim of this biomechanical investigation was to compare an angular stable locking plate using fenestrated, augmentable condylar screws vs. an angular stable locking plate with conventional, non-cemented condylar screws for the fixation of supracondylar femoral fractures.

Due to the screw design, it was hypothesized that the cementable condylar screws would provide a more accurate cement application without cement extrusions, leading to improved stability of the implant in specimens with reduced bone structure. Following this, it was hypothesized that the load to failure of osteosynthesis in the cement augmented group would increase.

## Methods

### Specimens

For this biomechanical study, sixteen femora originating from eight human beings were utilized. Written consent was obtained from the body donors who were provided by the local anatomical institute. A positive ethics approval was obtained by the local ethics committee (AZ 56/17) to ensure that this research complied with the Declaration of Helsinki as well. The femora were enveloped in humidified clothes using a liquid, consisting of 96% ethanol and < 2% formaldehyde. To prevent dehydration of the specimens, they were stored in a cold storage room [7].

### Assessment of bone quality

To scan for prior fractures, lytic bone lesions or other bone pathologies, conventional anteroposterior and mediolateral radiographs using a c-arm unit were carried out. Bone mineral density (BMD) was evaluated using Dual-energy X-ray Absorptiometry (DXA) imaging at the proximal femur.

### Implants and surgical treatment

The Non-Contact Bridging plate for Distal Femur (NCB®-DF, Zimmer-Biomet Inc., Winterthur, Switzerland) was used as implant for this biomechanical study. The NCB®-DF is an angular stable locking plate, which fits to the lateral cortex of the distal femur through its anatomic design. The surgical procedures were performed by two experienced surgeons (BM and BC) using exclusively NCB®-DF plates with a length of nine holes.

As an attempt to control for specimen variability it was decided to randomly assign all the right specimens to the augmented group. Thus, the left femur of each pair of bones was treated without bone cement.

**Fig. 2:**
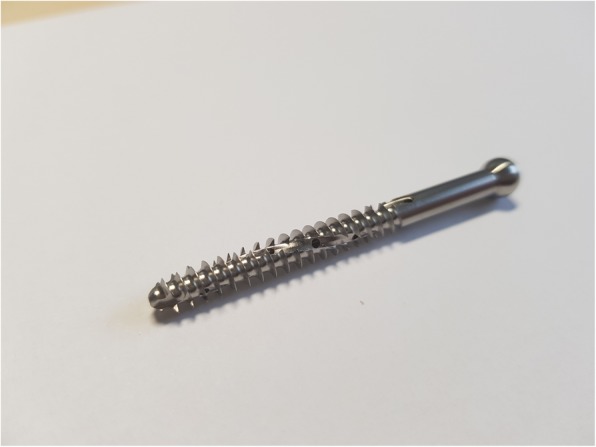
Illustration of the new, partially threaded, fenestrated, cancellous, condylar screw (diameter 5 mm) suitable for the NCB®-DF system. The screw provides two longitudinal furrows and ten perforation holes, for an optimal cement extrusion along the shaft of the screw

**Fig. 3:**
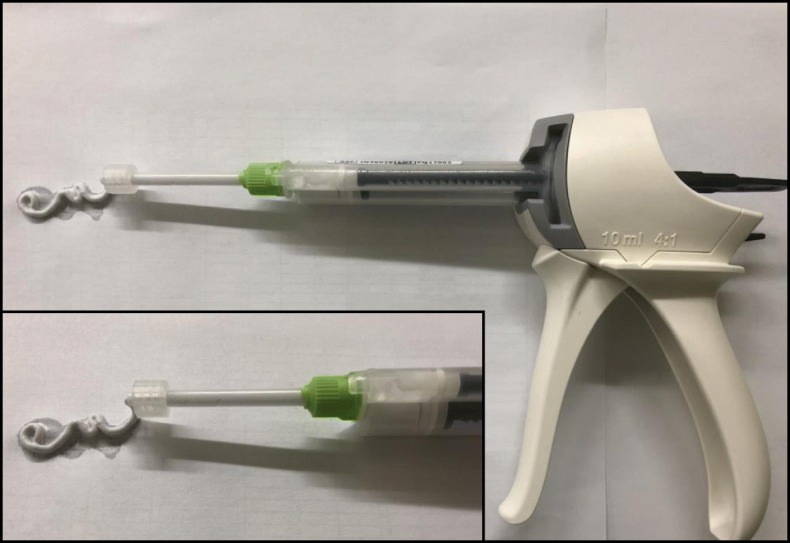
Cement injection gun (large picture detail) with cement applicator and iN3 calcium phosphate bone cement (small picture detail)

**Fig. 4:**
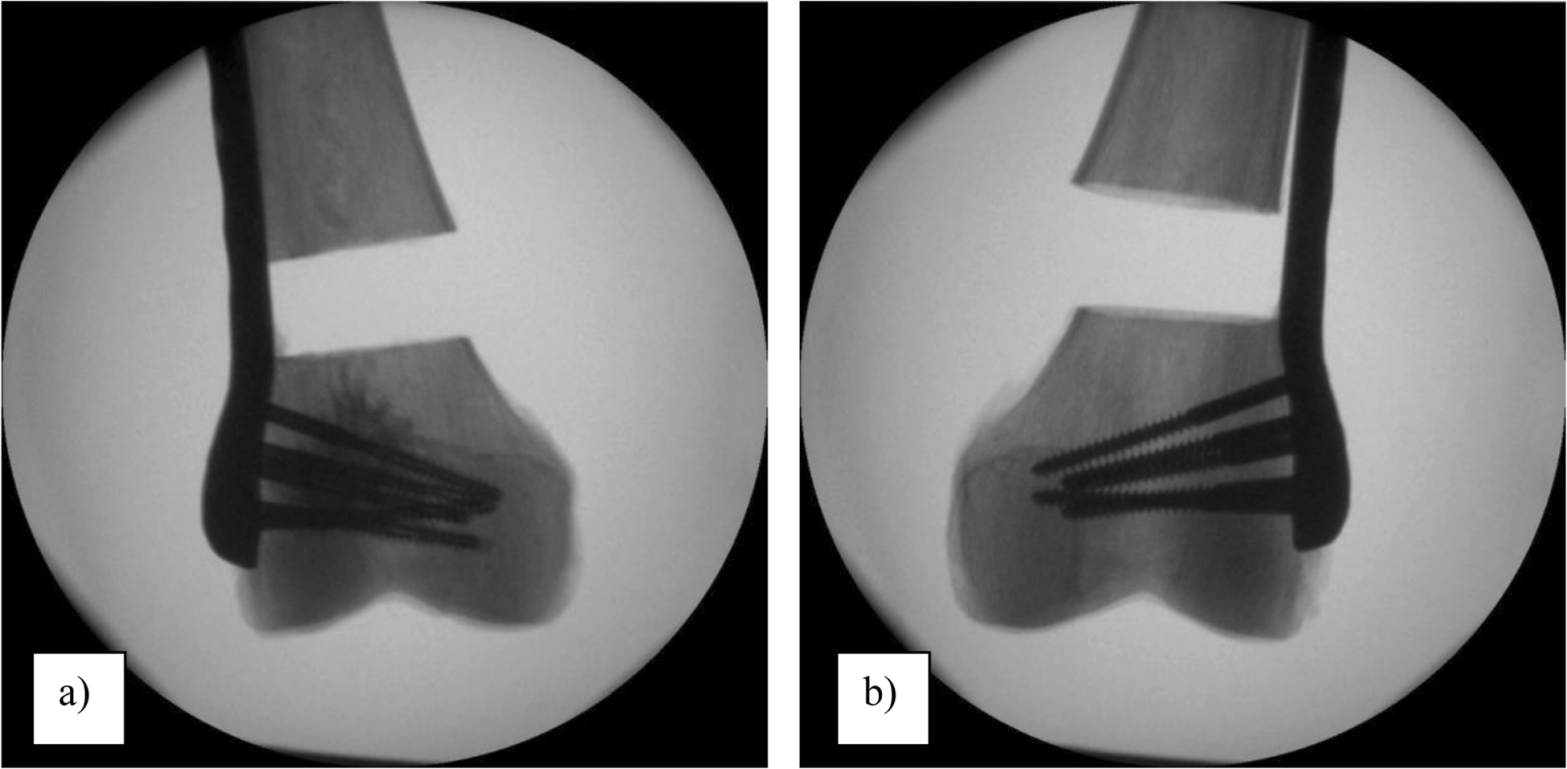
Typical postoperative X-ray pictures showing a pair of samples with cement augmented, fenestrated condylar screws in the right femur (**a**) and with non-augmented condylar screws in the left femur (**b**)

The specimens assigned to the augmented group received five newly designed, fenestrated, cancellous screws (diameter 5 mm) positioned with a 2.5 mm drill hole. These screws were positioned in the distal part of the NCB®-DF osteosynthesis (Fig. 2). In each fenestrated screw, 3 ml of pre-mixed calcium phosphate bone cement (CPC) (iN3 bone cement; CelgenTek, LTD, Shannon, Ireland) was injected using a bone cement injection gun and an applicator (Fig. 3). In the non-augmented group, all the left femura were supplied with five conventional cancellous condylar screws (diameter 5 mm), available for the NCB®-DF system. To ensure an exact fit of the osteosynthesis and correct positioning of the applied cement, all different steps of the surgery were performed under fluoroscopic control, without the occurrence of any cement leakage (Fig. 4a and b).

A standardized fracture was performed using a surgical saw. As a fracture model, an instable supracondylar femoral fracture was established classified as 33-A3 according to the AO/ OTA. Perpendicular to the femoral shaft, an osteotomy was performed simulating a comminuted fracture of 2 cm length. To determine the height of the osteotomy, the individual condyle width was measured at the level of the joint line. The osteotomy was than performed at 75% of the measured length above the joint line. Afterwards shaft fixation was performed with five cortical screws placed in the proximal part of the plate, once again under fluoroscopic assistance.

### Experimental setup and biomechanical testing

**Fig. 5:**
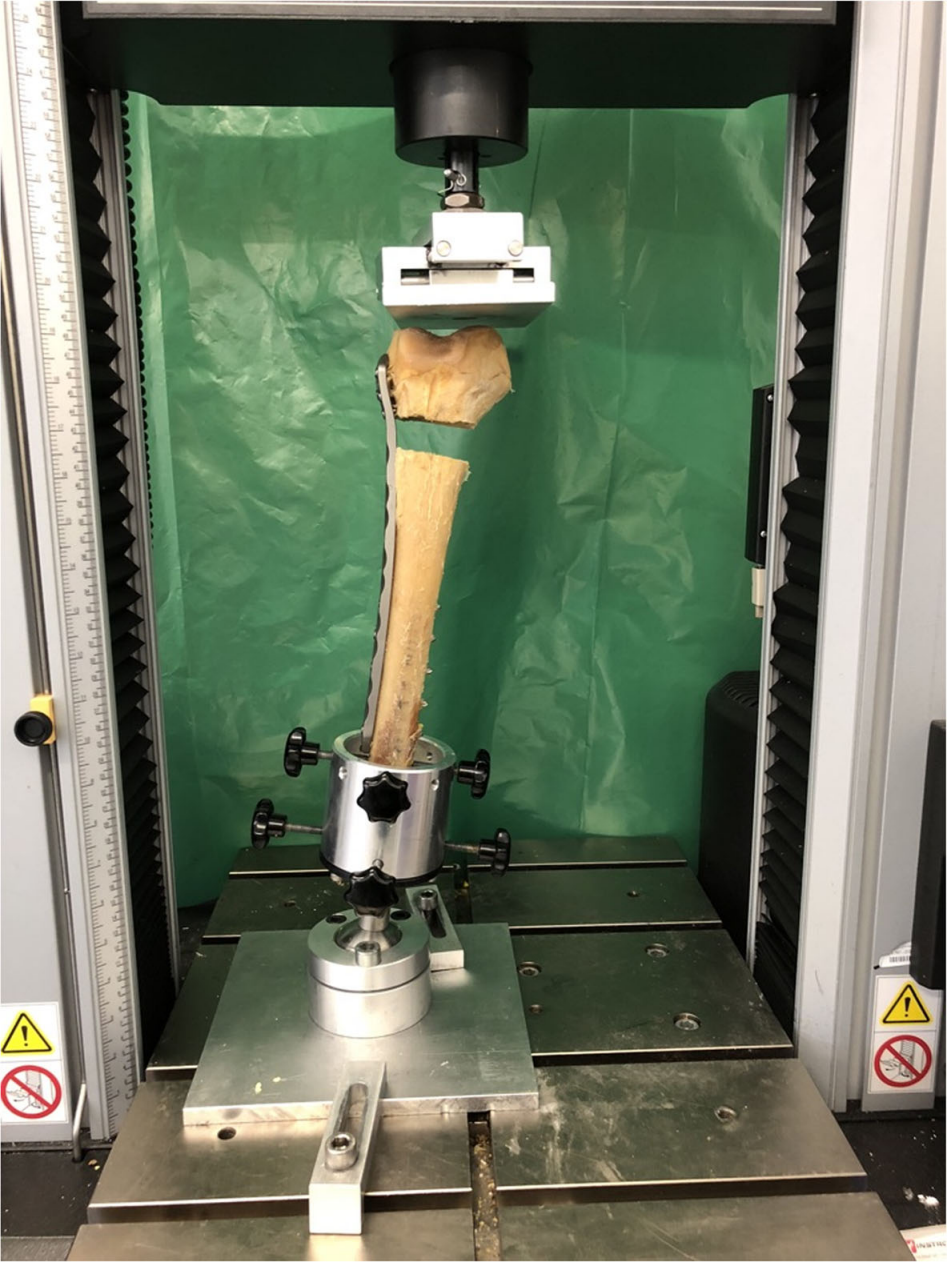
Test setup demonstrating force application with a metal plate, movable in two directions. The femoral shaft is statically fixed in an anatomical 5–7° valgus position

After the osteosynthesis, the proximal femora were resected with a surgical saw with the resection exceeding the proximal end of the osteosynthesis by 6 cm. Then the proximal shaft was grounded in a custom made form cup using Technovit 3040 (Heraeus, Wehrheim, Germany), a two-component synthetic substance. With a special holding device the femora were placed inversely in an Instron 5566 loading machine (Instron Cor., Darmstadt, Germany). By this means the femur condyles were placed horizontally and the weight bearing axis was in line with the femoral shaft (Fig. 5). A metal plate, mobile in more dimensions (anteroposterior and mediolateral) worked as pressure device.

A loading protocol, well known for the biomechanical analysis of supracondylar femoral fractures, was utilized [5]. Every sample was examined step by step with raising cyclic sinusoidal load. Pressure was transferred vertically through the femoral shaft.

A preload of 100 N was applied. Loading itself consisted of steps of 500 cycles, beginning with a load of 600 N. The testing machine stopped automatically at the end of each completed stress level. The load of each subsequent increment was raised by 200 N at a frequency of 1 Herz (HZ) until a failure of osteosynthesis occurred.

End points of the testing were breaking of the implant, bowing of the plate itself or significant loss of reposition due to cutting out of the screws out of the bone, respectively. Abort criteria for the biomechanical testing were a sudden load drop higher than 30% or an irreversible deformation of the specimen, higher than 20 mm. The examinations were performed in a compression set controlled mode.

### Data collection and statistical analysis

Data collection was done in 100 ms intervals based on the instrument-specific Bluehill software. Plastic deformation is defined as the extent of irreversible deformation after force application and was recorded using the maximum value in the last cycle. Loading (N), compression set (mm) and the number of cycles were recorded and given with a 95% confidence interval (CI). Stiffness of the constructs was calculated from as quotient between compression set and applied loading.

For the load to failure, as the primary outcome measure, a sample size of eight pairs of femora was calculated. A clinically relevant difference was defined if the failure occurred in 100% of non-augmented and up to 40% in augmented specimens at a load of 2000 N. The power was set to 0.80, with an alpha error of 0.05.

The IBM SPSS statistics package (IBM Corporation, Armonk, New York, USA) was applied. The Shapiro-Wilk test was used to test for normal distribution of the data. Depending on the outcome, a paired t-test or a Wilcox rank sum test for paired samples was performed. Statistical significance level was set at *p* < 0.05.

## Results

### Specimens

The bones originated from three male and five female adults, with an average age of 77 years (range 62–88 years). Dual-energy X-ray Absorptiometry (DXA) detected a compromised bone quality of all tested specimens with a mean T-score of − 2.82 (CI: − 3.33 - -2.31). The bone mineral density was comparable in all matched femora samples (*p* = 0.244).

Radiographic analysis prior to testing revealed no unexpected results like bone fissures or fractures. Postoperative radiologic examination showed a cutting out of the condylar screws in five specimens of the cement augmented group. Deformation by compression of the condylar bone stock was seen two times, and condylar fractures were seen once in cement augmented group.

**Fig. 6:**
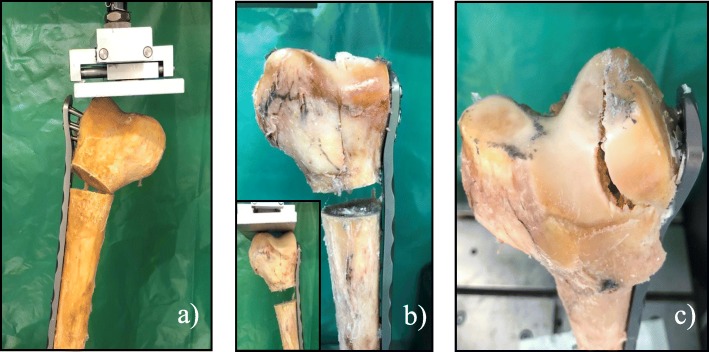
Photographs showing the different types of construct failures. Cutting out of the screws (**a**) was most common in both groups, followed by deformation of the condylar region (**b**). This deformation of the condylar region can be seen by comparing the position of the distal end of the plate in relation to the joint line before loading ((**b**) small picture) and at the end of the testing ((**b**) big picture). Condylar fracture (**c**) was only seen once in the group with cement augmented condylar screws

In the non-augmented group, the deformation with cutting out of the condylar screws was seen six times. Deformation of the condylar bone was seen twice, while no fracture of the condylar region occurred. Fig. 6 a, b and c show the corresponding clinical photographs.

### Load to failure analysis

**Fig. 7:**
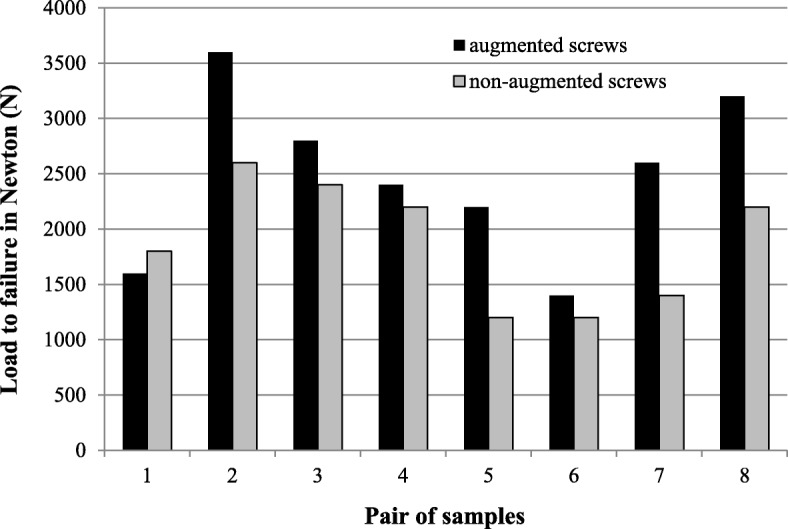
Failure loads based on the cyclic loading tests, comparing plate osteosynthesis with and without augmentation of the condylar screws

The mean compression force leading to failure was 1875 N (95% CI: 1320 N - 2430 N) in the non-augmented group and 2475 N (95% CI: 1727 N- 3223 N) in the group with augmented condylar screws. This difference was statistically significant (*p* = 0.024) (Fig. 7).

### Axial stiffness analysis

Due to the fact that at a load of 1200 N the first examined specimens collapsed, a stiffness analysis of the constructs was performed on a load level of 1000 N. At this load the stiffness was not significantly different between both groups (augmentation group: mean 3. 08 kN/mm; 95% CI: 1.49–4.66 kN/mm versus non-augmentation group: mean 2.79 kN/mm; 95% CI: 4.12–1.46 kN/mm; *p* = 0.889).

### Plastic deformation analysis

**Fig. 8:**
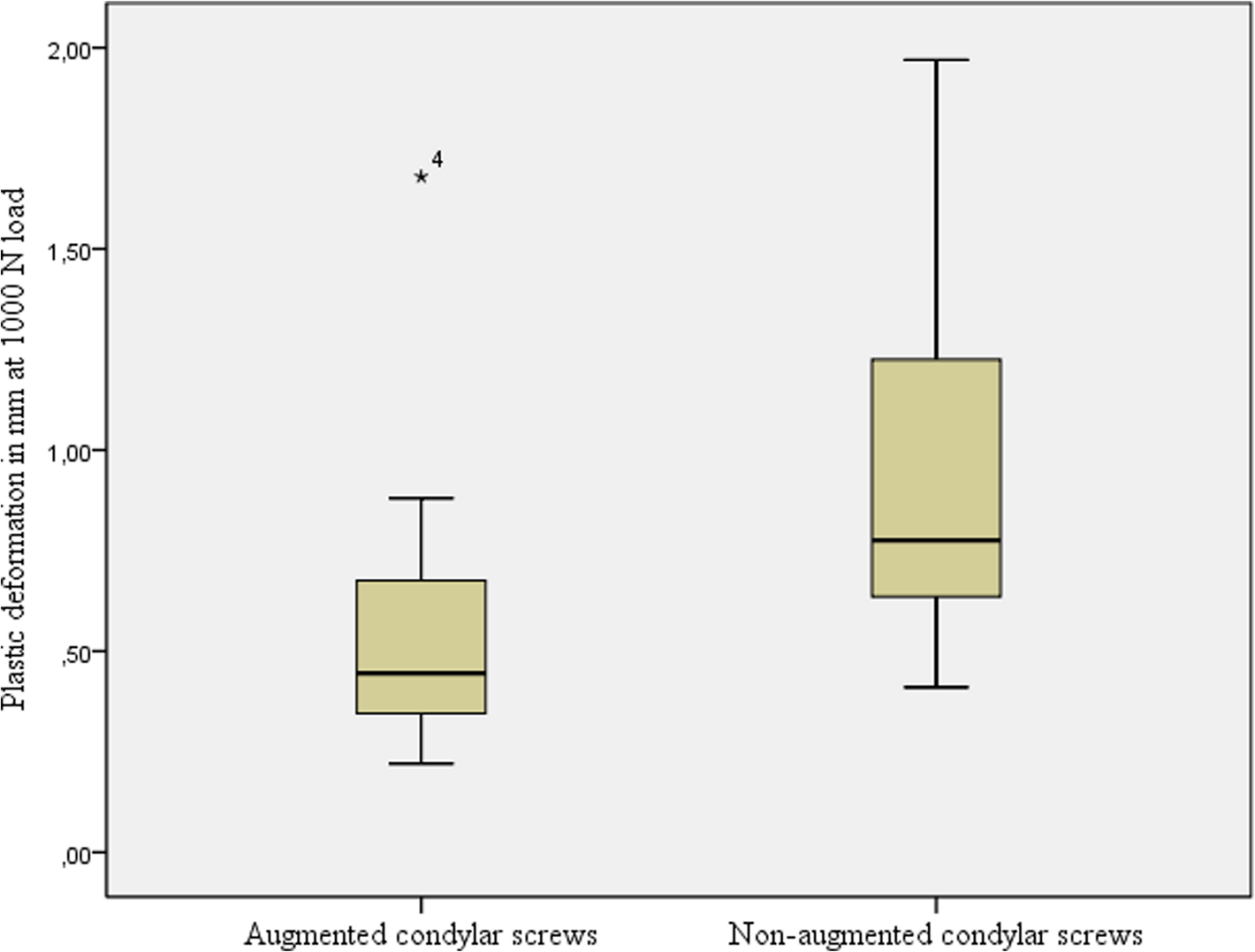
Plastic deformation of osteosynthesis with augmented condylar screws and non-augmented condylar screws at a load of 1000 N

Additionally plastic deformation of the osteosynthesis construct was analysed at the load level of 1000 N. As shown in Fig. 8, a higher variability in plastic deformation was revealed if osteosynthesis were conducted in the non-augmented group (mean 0,96 mm; 95% CI 0,41–1,50 mm) as compared to the augmented specimens (mean 0,60 mm; 95% CI 0,13–1,08 mm). Nevertheless, this difference in plastic deformation between both types of osteosynthesis failed to reach statistical significance (*p* = 0.161).

## Discussion

The reported biomechanical experiments analyzed the influence of implant augmentation for the surgical treatment of supracondylar femoral fractures using newly designed fenestrated condylar screws to fix polyaxial locking plates. The study revealed that cement augmentation of condylar screws lead to a significantly higher load to failure, whereas stiffness and plastic deformation did not show a significant difference between both groups.

Former biomechanical studies on fracture fixation in distal femoral fractures already showed the potential of polymethylmethacrylate (PMMA) cement for augmentation of condylar screws in osteoporotic bone [5]. Currently, cement-related problems concerning disturbances of the bone metabolism are discussed. The exothermic hardening of PMMA cement generates temperatures up to 45 °C and might increase the risk of bone and cartilage necrosis [8].

In this context, CPC overcomes the temperature-related drawbacks of PMMA cement, as CPC hardens within minutes after injection without generating heat [9]. IN3 is a mineral bone cement containing calcium and phosphate salts finely dispersed in a biocompatible lipid-based liquid. The cement hardens through the creation of hydroxyapatite crystals. The cement sets completely intra-operatively and maintains its shape with no volume loss or shrinkage. IN3 calcium phosphate-based cement remodels through cell-mediated processes. The cement is osteoconductive, and remodelling proceeds through a one-to-one substitution process with the surrounding bone.

Uncontrolled extrusion of cement e.g., into the surrounding soft tissue or into the joint line as well as pulmonary cement embolism into the lung, as already described in other fields of orthopaedic surgery, remain a further serious complication of cement augmentation of orthopaedic implants [10]. In this context, the introduction of fenestrated cancellous screws for implant augmentation is supposed to be a further innovation as compared previous research available on this topic. Regarding this the fenestrated cancellous screws, were designed with holes starting in the proximal part of the screw thread, to facilitate a preferably secure application of bone cement into the condylar region. Thus, a cement cloud could be established in the midshaft of the screws without any uncontrolled cement leakage into the joint line or through the medial cortex of the bone (Fig. 4a).

Cementation of the condylar screws resulted in a significant increase in the load to failure in the present biomechanical setting. It is presumed that such an increase in loading capacities is conditioned by an enlargement of the bone-implant interface of the cemented condylar screws, leading to an increased resistance in the osteoporotic bone [11].

Further study results revealed that the biomechanical characteristics of the augmented constructs have not been changed, as values for axial stiffness and plastic deformation remained equal between augmented a non-augmented specimens. Therefore the results of the present study are in line with previous biomechanical investigations on the augmentation of plate osteosynthesis in distal femoral fractures conducted by *Bliemel* et al. and *Wähnert* et al. [5, 6]. *Bliemel* et al. concluded that the majority of motion of the osteosynthesis most likely occurs through bending of the plate itself under the influence of axial force rather than through micromotion of the screws in the bone. The results of the present study also favour the assumption that the natural, biological performance of CPC, which is characterized by a high degree of biocompatibility and bioactivity, might have contributed to such comparable biomechanical characteristics [12, 13].

Even though the present biomechanical study tried to overcome the methodical drawbacks of former studies conducted on this topic, there remain some limitations.

In this context, it should be noted that the use of formaldehyde embalmed human cadaver bones might have affected the comparability of the present biomechanical results to actual failure modes in real life patients. In this context there are controversies in the current literature concerning the authenticity of loading tests performed with formaldehyde preserved cadaveric bones. While *Hammer* et al. and *Unger and co-workers* pointed out organic changes through the preservation process in cadaveric bones [14, 15], *Topp* et al. found no significant differences between fresh frozen and embalmed femora concerning pull-out forces of cancellous and cortex screws and axial load until failure [16].

Another putative limitation concerns the in vitro set up which only tested the axial loading of the specimen. The set up fails to test torsional and bending loads. Finally, with only eight femora the number of samples available for the test was rather small. However, a careful study design and the inclusion of a power analysis prior to the test should partially overcome these limitations. Future approaches will focus on an optimized screw design to further improve implant integrity.

## Conclusion

Based on the present study results, the newly introduced, cementable condylar screw could be an encouraging feature for the fixation of supracondylar femoral fractures in patients with reduced bone quality in terms of load to failure accuracy of the cement application.

## Data Availability

The corresponding author provides the data on demand.

## Abbreviations

CIs: Confidence intervals
CPC: Calcium phosphate bone cement
DXA: Dual-energy X-ray Absorptiometry
Hz: Herz
mm: Millimetre
ms: Milliseconds
N: Newton
NCB®-DF: Non-Contact Bridging plate for Distal Femur
PMMA: Polymethylmethacrylate
SPSS: Statistics Statistical Package for the Social Science
